# Copeptin as a Serum Biomarker of Febrile Seizures

**DOI:** 10.1371/journal.pone.0124663

**Published:** 2015-04-20

**Authors:** Benjamin Stöcklin, Sotirios Fouzas, Paula Schillinger, Sevgi Cayir, Roswitha Skendaj, Michel Ramser, Peter Weber, Sven Wellmann

**Affiliations:** 1 Division of Pediatric Neurology and Developmental Medicine, University of Basel Children’s Hospital (UKBB), Basel, Switzerland; 2 Department of Pediatrics, University Hospital of Patras, Patras, Greece; 3 Emergency Department, University of Basel Children’s Hospital (UKBB), Basel, Switzerland; 4 Department of Laboratory Medicine, University of Basel Hospital, Basel, Switzerland; 5 Division of Neonatology, University of Basel Children’s Hospital (UKBB), Basel, Switzerland; University of Rome Tor Vergata, ITALY

## Abstract

**Background and Objectives:**

Accurate diagnosis of febrile seizures in children presenting after paroxysmal episodes associated with fever, is hampered by the lack of objective postictal biomarkers. The aim of our study was to investigate whether FS are associated with increased levels of serum copeptin, a robust marker of arginine vasopressin secretion.

**Methods:**

This was a prospective emergency-setting cross-sectional study of 161 children between six months and five years of age. Of these, 83 were diagnosed with febrile seizures, 69 had a febrile infection without seizures and nine had epileptic seizures not triggered by infection. Serum copeptin and prolactin levels were measured in addition to standard clinical, neurophysiological, and laboratory assessment. Clinical Trial Registration: NCT01884766.

**Results:**

Circulating copeptin was significantly higher in children with febrile seizures (median [interquartile range] 18.9 pmol/L [8.5-36.6]) compared to febrile controls (5.6 pmol/L [4.1-9.4]; *p* <0.001), with no differences between febrile and epileptic seizures (21.4 pmol/L [16.1-46.6]; p = 0.728). In a multivariable regression model, seizures were the major determinant of serum copeptin (*beta* 0.509; *p* <0.001), independently of clinical and baseline laboratory indices. The area under the receiver operating curve for copeptin was 0.824 (95% CI 0.753-0.881), significantly higher compared to prolactin (0.667 [0.585-0.742]; *p* <0.001). The diagnostic accuracy of copeptin increased with decreasing time elapsed since the convulsive event (at 120 min: 0.879 [0.806-0.932] and at <60 min: 0.975 [0.913-0.997]).

**Conclusions:**

Circulating copeptin has high diagnostic accuracy in febrile seizures and may be a useful adjunct for accurately diagnosing postictal states in the emergency setting.

## Introduction

Febrile seizures (FS) occur in 2–5% of children between six months and five years of age and represent the most common convulsive event in childhood.[1] Although FS arise typically at the onset of fever accompanying otherwise uneventful infections,[2] it is often difficult to differentiate between FS and non-ictal events at the emergency setting, especially when history and clinical presentation are unclear.

Circulating prolactin may be elevated after paroxysmal epileptic events and, despite the uncertainty surrounding its diagnostic accuracy, it has been proposed as an adjunct to *post-facto* diagnosis when history of seizures is equivocal.[3,4] Another hormone released by the pituitary gland, arginin-vasopressin (AVP), has been shown to be involved in the thermoregulatory response to fever and convulsions;[5–8] recent evidence also suggests that AVP synthesis may be up-regulated in kainic acid-induced seizures in animal models.[9,10] Although, AVP is unstable in the peripheral blood and, therefore, unsuited for diagnostic use,[11,12] the C-terminal portion of the AVP precursor, copeptin, has been recognized as a robust marker of AVP secretion.[13] To date, there is a paucity of data in the literature regarding postictal serum copeptin levels in children with febrile or epileptic seizures (ES).

In the present study we hypothesized that serum copeptin may be elevated in children after FS, and that the release of the hormone may be related to the epileptic activity *per se* and not to the underlying infection. To test our hypothesis we prospectively investigated postictal serum copeptin levels in children with FS, in comparison to children with febrile infections without seizures and children with ES not triggered by infection. We also sought to compare the diagnostic accuracy of serum copeptin with that of circulating prolactin in the same cohort.

## Patients and Methods

### Study Design and Setting

This prospective, cross-sectional study (EpiCop) was conducted at the University Children’s Hospital of Basel (UKBB), Switzerland, between May 2013 and April 2014. The Cantonal Ethics Committee of Basel approved the study protocol (EK352/12), and written informed consent was obtained from the parents. The study was registered at Clinicaltrials.gov (No. NCT01884766).

### Patients

During the study period, children presenting at the emergency department (ED) for a convulsive event (associated or not with fever) were evaluated for eligibility based upon the following criteria: 1. Age between five months and six years. 2. Medical indication for blood sampling at the ED. 3. Parental consent to participate in the study. Eligible subjects were allocated into three groups: A) Febrile seizures group: Children with FS were children for whom parents or caregivers reported a convulsive event associated with elevated body temperature (>38°C) without a previous history of afebrile seizures. An experienced pediatric neurologist (PW) reviewed the medical files of those children and confirmed the diagnosis. FS were further categorized as simple or complex according to the standard criteria.[2] B) Febrile controls group: This group consisted of children presenting with a febrile infection and without previous history of febrile or afebrile seizures. Participants of this group served as the primary controls for testing the study hypothesis. None of the patients with fever (i.e., neither with FS nor controls) were septic or in septic shock. C) Epileptic seizures group: Children with ES were children with diagnosed epilepsy presenting for a convulsive episode not triggered by infection. Participants of this group served as secondary controls for assessing the differences in serum copeptin between FS and ES.

### Clinical Variables

The following parameters were recorded for each participant: sex, age, body weight, body temperature at home, body temperature and vital signs at the ED, clinical characteristics of the febrile infection, previous history of FS, time of onset, duration and characteristics of seizures, any relevant medication, and the amount of time between the episode and the presentation to the ED. Time of onset and duration of seizures were recorded according to the reports of parents. The laboratory testing included base analyses (hematocrit, white blood count with differential, serum sodium, and chloride), determination of C-reactive protein (CRP) and lactate, and blood gas analysis.

### Blood Sampling and Assays

For copeptin and prolactin measurements, at least 0.5 ml of whole blood was drawn in 1.2-ml Sarstedt (Nuembrecht, Germany) serum tubes, which were immediately send to the Department of Laboratory, centrifuged and the serum was frozen at −20°C immediately after collection. Copeptin measurements were performed using the BRAHMS Kryptor Compact immunoanalyzer (Thermo Scientific Brahms GmbH, Hennigsdorf, Germany), and prolactin measurements using the Roche Modular E 170 (Roche Diagnostics AG, Rotkreuz, Switzerland). For copeptin, the lower detection limit was 0.9 pmol/L, and the functional assay sensitivity (20% interassay coefficient of variance (CV)) 2 pmol/L. The inter-assay precision was < 7% CV at 5 pmol/L and < 4% CV at 100 pmol/L. For prolactin, the lower detection limit was 1 mU/L, and the inter-assay precision < 3% CV at 102, 450, and 816 mU/L, respectively. Both involved physicians at the ED and researchers were blinded to the biomarker levels until data analysis.

### Statistical Evaluation

Due to the paucity of data on copeptin levels in children or adults with seizures, *a priori* sample size calculation was not performed. However, a post-hoc power analysis (Wilcoxon-Mann-Whitney test) revealed that the present sample size was adequate to evaluate the observed differences in serum copeptin between children with FS and febrile controls at the 0.001 significance level with 88.1% power. Statistical power analysis was performed using the G*Power software.[14] Continuous variables were expressed as median with interquartile range (IQR) and compared using the Mann-Whitney U test. Simple and multivariable linear regression analysis was applied to explore the effect of FS, sex, age, body temperature at home or ED, and various laboratory parameters on (logarithmic-transformed) serum copeptin levels in children with febrile infections (i.e., excluding children with ES). A similar multivariable regression approach was used to explore the effect of various parameters on serum copeptin (separately for children with FS and those with fever without seizures). Spearman's rank order correlation was used to explore the relationship between serum copeptin and the time elapsed since the episode of seizures in children with FS. The discriminatory ability of both copeptin and prolactin was assessed by receiver operating characteristics (ROC) curve analysis and was compared by means of the area under the curve (AUC). All analyses were performed using the IBM SPSS software version 20.0 (IBM Corp., Armonk, NY). A p-value of < 0.05 was considered statistically significant.

## Results

During the study period, 158 children between six and 72 months of age presented at the ED due to seizures, either FS or ES. Of these, 41 were not possible to be evaluated for eligibility due to inconvenient time of presentation, 19 had no indication for blood sampling at the ED because of a simple FS with a previous history of uncomplicated FS, and in three cases parents refused to provide consent to participate. Eighty three children were allocated into the FS group. Twelve children were diagnosed with epilepsy; in nine of them, seizures were not triggered by infection and they were allocated into the ES group. According to the International League Against Epilepsy (ILAE) Commission on Classification and Terminology [15] the diagnoses of the ES patients were: generalized, genetic (n = 4); focal, secondarily generalized, structural (n = 2); focal, secondarily generalized, genetic (n = 1); focal, dyscognitive, structural (n = 1); focal, dyscognitive, genetic (n = 1). During the same period, a convenient sample of 69 children with febrile infections without seizures was included (primary control group). The characteristics of the three groups are presented in Table 1.

**Table 1:** Characteristics of study groups.

	Fever without seizures (n = 69)	Febrile seizures (n = 83)	Epileptic seizures (n = 9)
Males/Females	37/32	50/33	5/4
Age, years	2.0 (1.3–3.4)	1.6 (1.2–2.2) **	2.9 (2.7–4.7) **
Body weight, kg	12.4 (9.3–15.4)	12.0 (10.0–14.0)	15.0 (12.5–15.5)
Temperature at home, °C	39.9 (39.0–40.0)†	39.2 (39.0–40.0) †	NA
Temperature at ED, °C	38.4 (37.5–39.2)	38.8 (38.1–39.3)	NA
Duration of event, min	NA	3 (2–5)	5 (2–10)
Time to presentation, min	NA	90 (63–120)	75 (15–115)
Source of fever, n (%)			
URTI	42 (60.9)‡	68 (84.0) ‡	NA
LRTI	8 (11.6) ‡	0 (0.0) ‡	NA
UTI	4 (5.8)	1 (1.2)	NA
Gastroenteritis	7 (10.1)	5 (6.2)	NA
Other	6 (8.7)	2 (2.5)	NA
Unidentified	2 (2.9)	5 (6.2)	NA
Laboratory data*			
Hct, %	36.4 (33.4–38.5)	37.3 (34.2–39.8)	38.3 (35.8–40.8)
WBC, ×1000/mm^3^	11.7 (8.1–15.3)	11.3 (6.9–15.7)	7.9 (7.2–10.5)
Na, mmol/L	136 (134–139)	135 (134–137)	137 (136–138)
Cl, mmol/L	105 (103–107)	105 (103–106)	106 (105–107)
pH	7.43 (7.39–7.45)	7.40 (7.39–7.43)**	7.36 (7.33–7.39) **
CO_2_, mm Hg	32 (29–35)	33 (31–35)††	38 (36–41) ††
Base deficit, mmol/L	2.8 (0.9–4.5)	2.9 (2.0–3.7)	2.5 (1.8–4.5)
Lactate, mmol/L	1.2 (1.1–1.6)	1.3 (1.0–1.8)	1.2 (0.9–1.4)
CRP, mg/dl	31.0 (13.0–84.0)§	7.0 (0.3–19.0) §	0.3 (0.3–0.3)

Data are presented as median (IQR) unless stated otherwise.

*Blood analysis results at presentation.

^†^p <0.05,

^‡^p <0.01, and

^§^p <0.001 for comparisons between febrile seizures and controls.

^¶^p <0.05,

^**^p <0.01, and

^††^p <0.001 for comparisons between febrile and epileptic seizures.

Between-group comparisons were performed with Mann-Whitney U, chi square or Fisher's exact test as appropriate. ED: emergency department; URTI: upper respiratory tract infection; LRTI: lower respiratory tract infection; UTI: urinary tract infection.

Children with FS did not differ from febrile controls in terms of sex, age, body weight, body temperature at presentation, and laboratory base analyses (Table 1). Febrile controls exhibited higher body temperatures at home and higher CRP levels at presentation. In addition, the febrile controls were more frequently diagnosed with lower than upper respiratory tract infections (Table 1). Children with ES were older and exhibited lower blood pH and higher PaCO_2_ at presentation compared to participants with FS. The duration of seizures and the time elapsed since the episode was similar between children with FS and ES (Table 1). In total, 23 children (27.7%) in the FS group had a previous history of FS. The majority of FS (89.2%; n = 74) were simple, whereas the FS were characterized as complex in 20 cases (24.1%).

**Fig 1:**
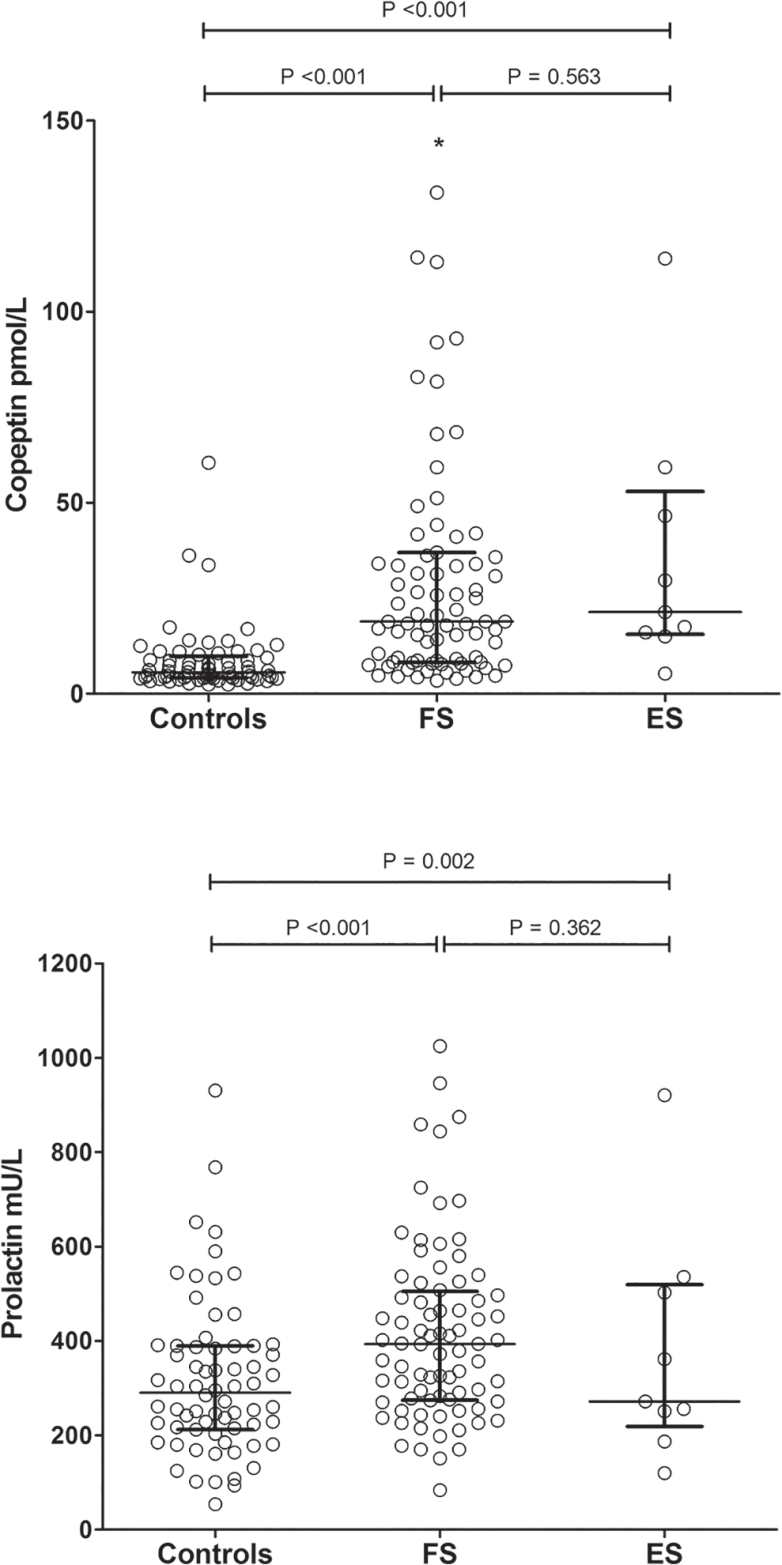
Scatter dot plots of serum copeptin and prolactin values in the three study groups. Medians and interquartile ranges are also presented. Between-group comparisons were performed with Mann-Whitney U test. * Serum copeptin values in 4 cases (range 208–306 pmol/L) are outside the copeptin-axis limits of the graph. FS: febrile seizures; ES: epileptic seizures

Serum copeptin levels were increased in children with FS (median 18.9 pmol/L, IQR 8.5–36.6 pmol/L) compared to febrile controls (median 5.6 pmol/L, IQR 4.1–9.4 pmol/L; *p* < 0.001); however, no differences between children with FS and ES were noted (median 21.4 pmol/L, IQR 16.1–46.6 pmol/L; *p* = 0.563) (Fig 1). Serum prolactin was also higher in children with FS compared to controls (median 394 mU/L, IQR 275–503 mU/L; median 291 mU/L, IQR 214–390 mU/L; respectively; *p* < 0.001), but no differences were detected between children with FS and ES (ES median 272 mU/L, IQR 251–503 mU/L; *p* = 0.362) (Fig 1).

Neither copeptin nor prolactin levels differed between children with simple or complex FS (copeptin: median 17.9 pmol/L, IQR 7.9–34.9 pmol/L vs. median 27.2 pmol/L, IQR 18.0–41.6 pmol/L, respectively, *p* = 0.090; prolactin: median 376 mU/L, IQR 263–468 mU/L vs. median 422 mU/L, IQR 323–551 mU/L, respectively, *p* = 0.197).

Multivariable regression analysis revealed that FS were the major determinant of serum copeptin levels in febrile children (regression coefficient *beta* 0.509, *p* < 0.001) independent of sex, age, body weight, body temperature at home or at presentation, laboratory base analyses, and CRP levels (Table 2). Relative blood acidosis, in terms of a higher base deficit (*beta* 0.216, *p* = 0.011) or lower blood pH (*beta* -0.173, *p* = 0.044), was also a significant and independent predictor of serum copeptin levels in that cohort (Table 2). When children with FS were analyzed in a separate multivariable regression model, serum copeptin levels were related to the time elapsed since the seizure episode (*beta* -0.306, *p* = 0.004) and—to a lesser extent—to serum sodium (*beta* 0.204, *p* = 0.049) (S1 Table). Spearman's rank correlation analysis confirmed the inverse relationship between copeptin levels and the time elapsed since the event (*rho* -0.483, *p* < 0.001) (S1 Fig). In febrile controls, a higher base deficit was the only significant and independent predictor of serum copeptin levels (*beta* 0.396, *p* = 0.007) (S1 Table).

**Table 2:** Copeptin dependencies in febrile children with or without seizures.

	Unadjusted effect			Adjusted effect			
	R^2^	Beta	p-value	Model 1 (R^2^ 0.352)		Model 2 (R^2^ 0.368)	
N = 152*				Beta	p-value	Beta	p-value
Febrile seizures	0.287	0.536	<0.001	0.478	< 0.001	0.509	< 0.001
Male gender	0.006	0.078	0.322	-	-	-	-
Age	0.035	- 0.188	0.020	- 0.113	0.187	- 0.113	0.183
Body weight	0.024	- 0.156	0.054	-	-	-	-
Temperature at home	0.026	- 0.161	0.076	- 0.052	0.547	- 0.066	0.442
Temperature at ED	0.001	0.011	0.888	-	-	-	-
Hct	0.058	0.240	0.003	0.147	0.090	0.124	0.146
WBC	0.004	0.063	0.482	-	-	-	-
Na	0.001	0.003	0.976	-	-	-	-
Cl	0.002	0.049	0.586	-	-	-	-
pH	0.051	- 0.227	0.011	- 0.173	0.044	-	-
CO_2_	0.005	0.067	0.456	-	-	-	-
Base deficit	0.040	0.200	0.025	-	-	- 0.216	0.011
Lactate	0.002	0.043	0.638	-	-	-	-
CRP	0.053	- 0.231	0.009	0.037	0.695	0.039	0.683

Unadjusted and adjusted effect of each factor was calculated by simple and multivariable linear regression analysis using serum copeptin values (after logarithmic transformation) as the dependent variable. Only factors with statistically significant unadjusted effects were considered for the adjusted models.

* children with epileptic seizures are excluded.

**Fig 2:**
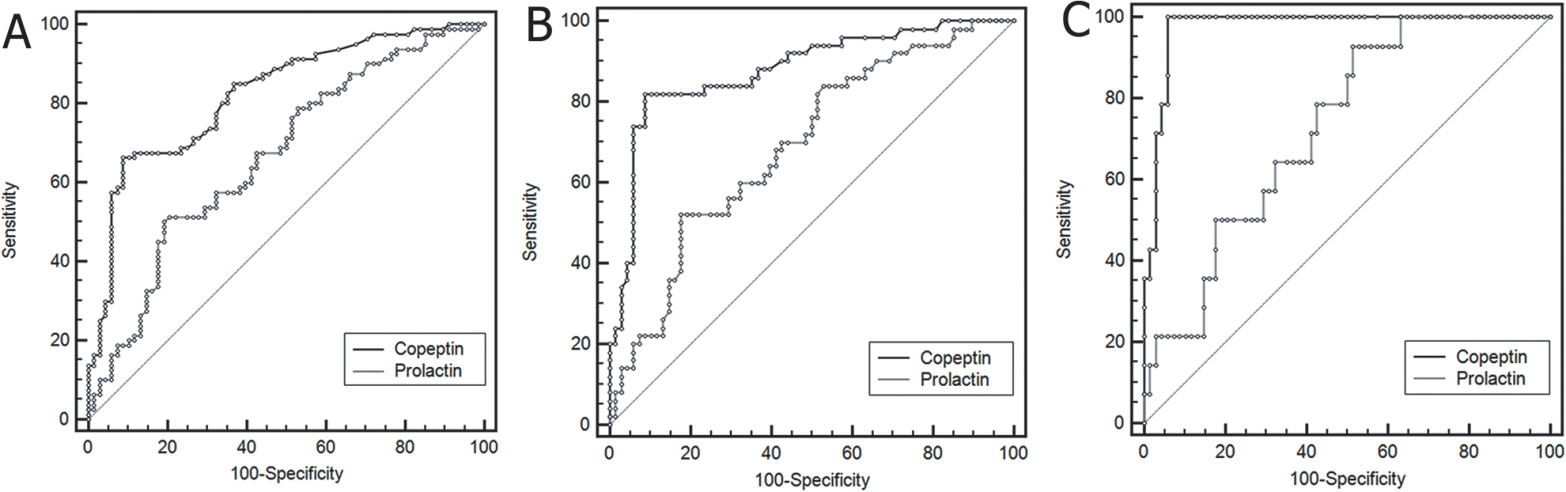
Receiver operating characteristic curves of serum copeptin and prolactin. (A) In controls (n = 69) and in children with febrile seizures irrespectively to the time of presentation (n = 83); (B) In controls (n = 69) and in children with febrile seizures presented <120 minutes since the event (n = 53); (C) In controls (n = 69) and in children with febrile seizures presented <60 minutes since the event (n = 14).

ROC curve analysis revealed that copeptin exhibited an increased overall ability to differentiate between children with FS and controls compared to prolactin (AUC 0.824, 95% CI 0.753–0.881 vs. 0.667, 95% CI 0.585–0.742; *p* <0.001) (Fig 2A). The discriminatory ability of copeptin was enhanced when only children with FS who presented within 120 minutes from the event were included in the analysis (AUC 0.879, 95% CI 0.806–0.932). In that case, the diagnostic performance of copeptin was also superior to prolactin (AUC 0.690, 95% CI 0.598–0.772; *p* < 0.001) (Fig 2B). Serum copeptin exhibited the maximum diagnostic ability when only children with FS who presented within 60 minutes from the event were analyzed (AUC 0.975, 95% CI 0.913–0.997), which was again significantly higher as compared to prolactin (AUC 0.729, 95% CI 0.620–0.821; *P* < 0.001) (Fig 2C). A serum copeptin threshold of 13 pmol/L was identified as having the optimal combination of sensitivity and specificity to differentiate between children with or without FS (Table 3).

**Table 3:** Diagnostic performance of selected copeptin cut-off values.

Copeptin (pmol/L)	Sensitivity (%)	Specificity (%)	Positive LR	Negative LR
> 4.0	97.6	23.2	1.3	0.10
> 6.0	90.4	53.6	1.9	0.20
> 13.0*	67.5	88.4	5.8	0.37
> 17.0	56.6	94.2	9.8	0.46

* optimal criterion according to the sum of sensitivity and specificity.

## Discussion

In this study, we prospectively investigated postictal serum copeptin levels in children with FS, in comparison to febrile children without seizures and children with ES not triggered by infection. We found that serum copeptin was significantly higher in children with FS as compared to febrile controls. The accuracy of copeptin in diagnosing FS was excellent within the first hour from the paroxysmal event and overall significantly higher to that of prolactin. These findings suggest that copeptin may be a promising new blood marker to adjust the diagnosis of FS at the emergency setting, when history and clinical presentation are equivocal.

Among the various clinical and biochemical variables recorded in this study, univariate regression analysis revealed an association between copeptin levels and febrile seizures, age, hematocrit, pH, base deficiency, and CRP (Table 2). However, in multivariable models only febrile seizures, pH, and base excess were proven significant (Table 2). Respiratory alkalosis has been reported to be involved in hyperthermia-induced FS in animal models [16] and was found to occur in children with FS.[17] Furthermore, administration of 5% carbon dioxide has been proven a fast and potent anticonvulsant.[18] In a systematic investigation of various stressors using an infant ventilation model, we previously showed that respiratory alkalosis (but not acidosis) may increase copeptin levels in the peripheral blood.[19] At a first glance, the results of the present study do not support a link between FS, respiratory alkalosis, and copeptin release, since serum copeptin levels were related to a relative metabolic acidosis (in terms of lower pH and higher base deficit) (Table 2). However, blood sampling in our cohort was performed not at the onset of seizures but-on average- more than one hour later, when children were presented at the ED. That amount of time was almost twice as long as in the study reporting an association between respiratory alkalosis and FS.[17] Therefore, it is possible that the observed relative metabolic acidosis was in fact the compensatory response to a primary acid-base disturbance (i.e., respiratory alkalosis) which occurred prior or at the time of the convulsive event. In addition to these systemic changes, in-vivo experiments have shown that epileptic activity may decrease the intracellular and extracellular pH, and that the latter may persist up to one hour after the arrest of seizures.[20]

Increased serum copeptin levels have been also reported after short hypoxic events,[19] which are not uncommon during convulsive episodes; central cyanosis is one of the most frequently reported signs by parents and caregivers of children with FS. An acute drop in blood pressure during the paroxysmal episode might also trigger copeptin release, in a way similar to that proposed for copeptin increase in elderly patients with syncope.[21] Finally, increased copeptin during FS may be the result of unspecific stress, since the serum levels of the hormone have been shown to be related to the individual stress level.[22]

In our study, a number of children with FS had low copeptin levels (Fig 1) which were in the range of copeptin values reported in healthy preschool children.[23] It has been also reported that, irrespective of age, circulating copeptin levels are higher in healthy men as compared to women.[24,25] In our study no sexual disparity of serum copeptin was found. Whereas the duration of seizures was not related to copeptin levels, the time elapsed since the convulsive event was an independent predictor of serum copeptin concentration (S1 Table). Given that the half-life of copeptin in the peripheral blood is approximately 45–60 min,[19][24] the time delay between the episode of seizures and blood sampling might explain the lower copeptin values in those children. In line to the above hypothesis, our data showed that the diagnostic accuracy of copeptin increases with decreasing time elapsed since the convulsive event (Fig 2). On the other hand, relatively high copeptin values (i.e., higher than the 75^th^ control-group percentile) were noted in a small subset of the febrile controls (n = 8) (Fig 1). Seven of those children suffered from lower respiratory tract infections, which are known to increase serum copeptin levels.[23]

Serum prolactin levels have been suggested as an adjunct in the *post-facto* diagnosis of convulsive events in children.[4] However, the diagnostic accuracy of the hormone in our study was proven limited, even within the first hour from the episode of seizures. Therefore, in line with previous reports,[3] our findings do not support the routine use of prolactin for diagnosing the postictal state in the emergency setting. Heart failure biomarkers, such as B-type natriuretic peptide (BNP) and its precursor N-terminal pro BNP (NT-proBNP), have been also found to be elevated in children with tonic-clonic seizures (including FS), pointing towards an involvement of the neuro-cardio-endocrine axis in such events.[26,27] However, since NT-proBNP values may also increase in children with simple febrile infections,[28] these biomarkers seem less suited for accurately diagnosing FS at the emergency setting.

The following limitations must be considered. First, due to the cross-sectional design of our study, the primary control group consisted of children with more severe infections, as indicated by their significantly higher CRP values (Table 1). This can be explained by the fact that a blood puncture is usually not needed in children presenting with uneventful febrile infections, especially at their onset. Given that copeptin is elevated in children with pneumonia and sepsis,[23,29] it is likely that copeptin levels in the control group are higher than those one would expect for healthy subjects. Second, our results might be biased by the absence of a clear—clinical or biological—indicator of seizures. In our cohort, the majority of seizures had already ceased by the time of presentation, either due to self-limitation or because anticonvulsants had been given. Therefore, the diagnosis was solely based on history and clinical presentation and, thus, the possibility that cases with fever shivering were falsely considered as FS cannot be excluded.

Our study did not address the underlying mechanisms by which serum copeptin increases during seizures. In addition, our data did not reveal any difference in copeptin values with respect to the clinical classifications of FS. However, in addition to the herein described diagnostic value, it would be of great interest to determine whether copeptin provides new prognostic avenues for FS. Even though a single uneventful episode of FS is of less clinical and prognostic importance, the assessment of children at the emergency setting is often challenging even for experienced physicians, because the differential diagnosis is difficult.[30] Thus, in clinically equivocal situations, especially when history and clinical presentation are unclear, copeptin holds promise as a postictal biomarker at triage. Copeptin results are available in principal within less than one hour, including blood sampling, pre-analytics, and main analysis; such a short turnaround time is a crucial prerequisite for a biomarker in the emergency department.[13] Future studies should also address the relevance of copeptin in patients presenting with ES. Since the accurate diagnosis of paroxysmal events in both children and adults is hampered even when rigid diagnostic standards are applied,[31] copeptin might be a valuable adjunct to decrease the rate of misdiagnosis in epilepsy at the emergency setting.

In conclusion, this study presents the first data on serum copeptin levels in FS and points to copeptin as a promising new blood marker to adjust the diagnosis of FS when history and clinical presentation are equivocal. Future research might address whether copeptin may distinguish between certain epileptic syndromes or whether the magnitude of copeptin increase is related to clinical outcomes, namely the recurrence of FS and the risk to develop epilepsy.

## Supporting Information

S1 Table Copeptin dependencies in children with febrile seizures and in children with fever without seizures. Unadjusted and adjusted effect of each factor was calculated by simple and multivariable linear regression analysis using serum copeptin values (after logarithmic transformation) as the dependent variable. Only factors with statistically significant unadjusted effects were considered for the adjusted model.
(DOC)

S1 Fig Serum copeptin concentrations in relation to time since event. (TIF)
