# Supplementary material for: Copeptin as a Serum Biomarker of Febrile Seizures
Source: PLoS One. 2015 Apr 20;10(4):e0124663. doi: 10.1371/journal.pone.0124663 (PMC4404343; doi:10.1371/journal.pone.0124663)
Supplement: S1 Table — Unadjusted and adjusted effect of each factor was calculated by simple and multivariable linear regression analysis using serum copeptin values (after logarithmic transformation) as the dependent variable. Only factors with statistically significant unadjusted effects were considered for the adjusted model. (DOC) [file pone.0124663.s001.doc]

| S1 Table  Copeptin dependencies in children with febrile seizures and in children with fever without seizures | | | | | | | | | | | | | |
| --- | --- | --- | --- | --- | --- | --- | --- | --- | --- | --- | --- | --- | --- |
|  | Febrile seizures | | | | | |  | Fever without seizures | | | | | |
|  | Unadjusted effect | | |  | Adjusted effect (R^2^ 0.244) | |  | Unadjusted effect | | |  | Adjusted effect (R^2^ 0.184) | |
|  | R^2^ | Beta | p-value |  | Beta | p-value |  | R^2^ | Beta | p-value |  | Beta | p-value |
|  |  |  |  |  |  |  |  |  |  |  |  |  |  |
| Male gender | 0.017 | 0.130 | 0.243 |  | - | - |  | 0.009 | - 0.94 | 0.443 |  | - | - |
| Age | 0.081 | - 0.285 | 0.009 |  | - 0.190 | 0.253 |  | 0.007 | 0.083 | 0.498 |  | - | - |
| Body weight | 0.050 | - 0.224 | 0.042 |  | - 0.040 | 0.807 |  | 0.001 | 0.001 | 0.999 |  | - | - |
| Duration of event | 0.032 | 0.179 | 0.105 |  | - | - |  | - | - | - |  | - | - |
| Time elapsed since event | 0.113 | - 0.336 | 0.002 |  | - 0.306 | 0.004 |  | - | - | - |  | - | - |
| Temperature at home | 0.008 | - 0.091 | 0.481 |  | - | - |  | 0.001 | - 0.033 | 0.804 |  | - | - |
| Temperature at ED | 0.056 | - 0.237 | 0.031 |  | - 0.193 | 0.068 |  | 0.004 | 0.060 | 0.625 |  | - | - |
| Hct | 0.017 | 0.130 | 0.243 |  | - | - |  | 0.070 | 0.264 | 0.033 |  | 0.100 | 0.483 |
| WBC | 0.023 | 0.153 | 0.213 |  | - | - |  | 0.001 | - 0.014 | 0.918 |  | - | - |
| Na | 0.053 | 0.229 | 0.041 |  | 0.204 | 0.049 |  | 0.022 | - 0.150 | 0.321 |  | - | - |
| Cl | 0.015 | 0.122 | 0.282 |  | - | - |  | 0.001 | - 0.010 | 0.945 |  | - | - |
| pH | 0.026 | - 0.162 | 0.154 |  | - | - |  | 0.078 | - 0.279 | 0.061 |  | - | - |
| CO_2_ | 0.004 | 0.065 | 0.569 |  | - | - |  | 0.049 | - 0.221 | 0.139 |  | - | - |
| Base deficit | 0.024 | 0.156 | 0.170 |  | - | - |  | 0.174 | 0.418 | 0.004 |  | 0.396 | 0.007 |
| Lactate | 0.017 | 0.131 | 0.256 |  | - | - |  | 0.008 | - 0.088 | 0.562 |  | - | - |
| CRP | 0.005 | - 0.073 | 0.549 |  | - | - |  | 0.009 | 0.095 | 0.481 |  | - | - |
| Unadjusted and adjusted effect of each factor was calculated by simple and multivariable linear regression analysis using serum copeptin values (after logarithmic transformation) as the dependent variable. Only factors with statistically significant unadjusted effects were considered for the adjusted model. | | | | | | | | | | | | | |
