# Supplementary figures and images for: Copeptin as a Serum Biomarker of Febrile Seizures
Source: PLoS One. 2015 Apr 20;10(4):e0124663. doi: 10.1371/journal.pone.0124663 (PMC4404343; doi:10.1371/journal.pone.0124663)

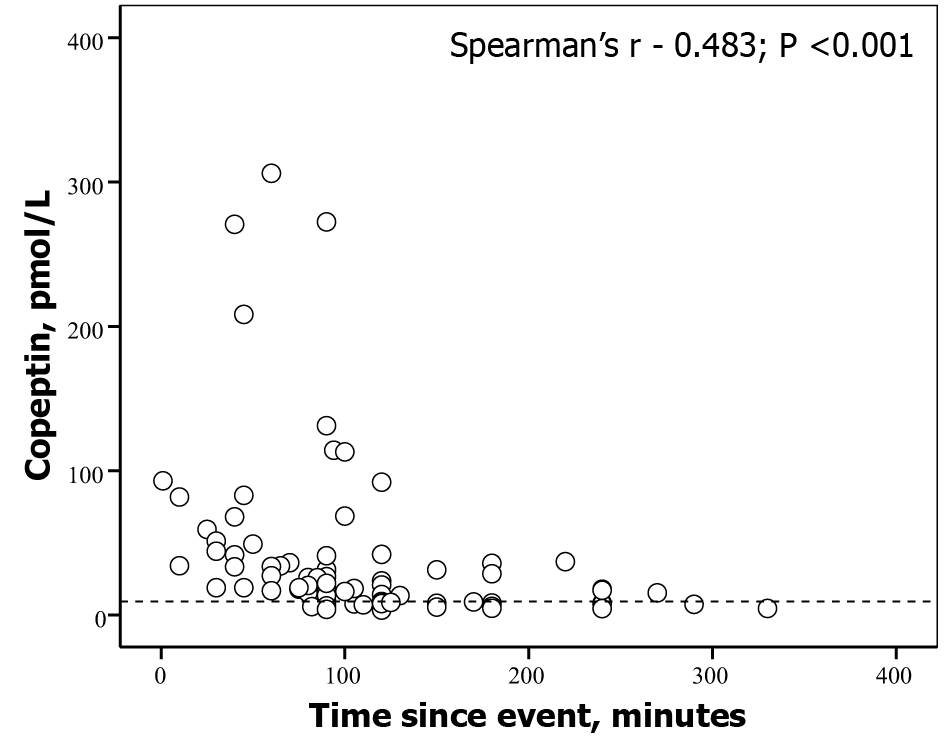

Supplement: S1 Fig — (TIF) [file pone.0124663.s002.tif]
